# Effects of Prenatal Lipopolysaccharide Exposure on Reproductive Activities and Serum Concentrations of Pituitary-Gonadal Hormones in Mice Offspring

**Published:** 2012-06-19

**Authors:** Jalal Solati, Ramin Hajikhani, Behnam Rashidieh, Mahshid Fatipour Jalilian

**Affiliations:** Department of Biology, Islamic Azad University, Karaj Branch, Karaj, Iran

**Keywords:** Lipopolysaccharides, Testostrone, Reproductive Activity, Prenatal, Mice

## Abstract

**Background:**

Maternal infection during pregnancy is a risk factor for some behavioral problems with neurodevelopmental origin. This study aimed to evaluate the effects of exposure of pregnant mice to the bacterial lipopolysaccharide (LPS) on sexual behaviour and serum level of pituitary-gonadal hormones of offspring in adulthood.

**Materials and Methods:**

In this Expremental study, pregnant NMRI mice (n=7/group) were treated with intra-peritoneal administration of LPS (1, 5 and 10 µg/kg) at day 10 of gestation. Induction of the pro-inflammatory cytokines, Tumor necrosis factor-alpha (TNF-α), interleukin-1beta (IL-1β) and interleukin-6 (IL-6) were measured in maternal serum 2 hours following the maternal LPS challenge. Behavior in the adult male offspring reproductive activity was investigated using receptive female mice. Concentrations of testosterone, luteinizing hormone (LH) and follicle-stimulating hormone (FSH) in adult offspring serum were measured using the enzyme-linked immunosorbent assay (ELISA) method (at postnatal day 60, n=10/group).

**Results:**

One-way ANOVA showed that LPS administration induces a significant increase in TNF-α, IL-1β and IL-6 levels of maternal serum. Prenatal LPS exposure reduces sexual behavior and serum concentration of LH and testosterone in adult male offspring.

**Conclusion:**

The overall results suggest that prenatal exposure to LPS increases pro- inflammatory cytokine levels, affects development of neuroendocrine systems and results in the inhibition of reproductive behaviors and reactivity of hypothalamic–pituitary-gonadal (HPG) axis in adult male offspring.

## Introduction

Previous studies have shown that lipopolysaccharide (LPS) affects brain development and results in behavioral disorders in many species. Epidemiological researches have reported that maternal bacterial and viral infections during pregnancy represent a risk factor for several neuropsychiatric disorders with a presumed neurodevelopmental origin. Studies using animal models have also shown that both bacterial and viral infections in utero can cause a spectrum of neuropathological and behavioral abnormalities in offspring (1-3).

Previous studies have shown that exposing pregnant female mice to stressors during the last week of pregnancy, reprograms the hypothalamicpituitary- adrenal (HPA) axis and enhances behavioral responses to psycho stimulants (4, 5).

Repeated exposure of pregnant mice to stressful environments during pregnancy has been found to induce deficits in cognitional behaviors (4). Moreover, increased risk for cognitive disorders have also been associated with maternal bacterial infections such as pneumonia during pregnancy (6, 7). The wide range of bacteria-related infections have been associated with increased risk for neurodevolepmental disorders, suggesting the common mechanisms to various prenatal infections that affect fetal development (6, 8).

Since previous studies considered, bacterial infections during pregnancy as a risk factor for brain and behavioral development in the fetus, this current study aims to investigate the effects of prenatal exposure to bacterial LPS on the development of reproduction-related behaviors and serum concentration of luteinizing hormone (LH), Follicle-stimulating hormone (FSH) nd testosterone in adult male offspring.

## Materials and Methods

### Animals and maintenance

In this experimental study, female and male NMRI mice obtained from Pasteur Institute of Iran, were aged between 10-12 weeks at the time of testing. Animals were housed in groups of 4 per cage in a room with a 12:12 hour light/dark cycle (lights on 07:00 am) under a controlled temperature (23 ± 1˚C). Animals had access to food and water. Breeding began after 2 weeks of acclimatization to the new animal holding room. The breeding procedure and the verification of pregnancy have been fully described in previous studies (1, 9). All of the pregnant rats were allowed to give birth and nurture their offspring normally. Littermates of the same sex were kept in groups of three to five mice. For standard milk availability, number of animals in each litter was standardized (3 males and 3 females/dam).

Pups were weaned on postnatal day 21 (PD 21), and offspring housed (four animals from the same treatment/cage) and maintained on a standard animal house condition. Adult male offspring randomly chose for each test were distributed into control and experimental groups (n=10/group, two pup each litter for the adulthood behaviors) before starting each test (10, 11).

All animal experiments have been carried out in accordance with the National Institutes of Health guide for the care and use of Laboratory animals (NIH).

### Treatments

In order to model a physiological maternal infection, at the 10th gestational day, pregnant mice were administered with intra-peritoneal injection of low doses of LPS (from Brucella abortus, Pasteur Ins., Tehran) which have been shown to produce optimal fever and cytokine induction in the mice, while having limited impact on maternal and pup survival (i.e. 1, 5 and 10 μg/kg of LPS, N=7/group) (6, 12-15).

### Serum cytokines assay

Maternal serum was prepared 2 hours after injection of saline or LPS by centrifugation at 15000 g for 5 min, aliquoted and then stored at -80˚C until the cytokine assays were performed. Concentrations of), interleukin-1beta (IL-1β) (Immuno-Biological Laboratories, IB49700, USA), interleukin-6 (IL-6) (BioSource International, CA 93012, USA), and Tumor necrosis factor-alpha (TNF-α) (Ucytech, CT 302, Netherlands) were determined using commercial enzyme-linked immunosorbent assay (ELISA) kits in accordance with the manufacturer’s instructions. All samples and standards were assayed in duplicate.

### Hormone assay

Testosterone, LH and FSH hormones of adult male offspring were assayed by solid phase ELISA kits (Demeditec Diagnostics Ltd., Germany), based on the principle of competitive binding and according to the manufacturer’s instruction.

The microtiter wells are coated with an antibody directed towards a unique antigenic site on the hormone molecule. Endogenous hormone of a serum sample competes with a hormone horseradish peroxidase conjugate for binding to the coated antibody. After incubation the unbound conjugate is washed off. The amount of bound peroxidase conjugate is proportionally reverse the concentration of hormone in the sample. After addition of the substrate solution, the intensity of color developed is proportionally reverse the concentration of hormone in the serum sample.

### Experiment layout

#### Reproductive activity tests in the male offspring

Adult male offspring were randomly chosen for behavioral testing. Receptive female mice were used to test male reproductive activity (Sniffing, Following, Mounting, Coupling) in such a way that males were placed in the female’s acrylic cage (25 cm×25 cm×40 cm; L×W×H) containing wood chips with food and water provided.

Before studying sexual behavior, control (prenatally exposed to saline) and LPS treated (prenatally exposed to LPS) male offspring were separately placed in a cage with a sexually experienced male and a receptive female to have prior learning or experience. Early morning of assessing day, sexually naive males were separated and maintained separately until that evening. Every naive male, currently sexually experienced, was given 60 minutes to accompany a receptive female, during which male behaviors were assessed and compared (16). Four separate replications of the experiment were run for each male. Sniffing, following, mounting and coupling were the sexual behavior parameters assessed. During 30-minutess sexual behavior tests if the male mice showed no mounts, the mounting component was over, and if not they were further permitted up to 60 minutes for coupling and ejaculation (16-18). During all testing sessions behavioral parameters were recorded on videotape and analyzed after completing the experiments (19).

### Statistical analysis

Since data displayed normality of distribution and homogeneity of variance, one-way ANOVA and Tukey Post Hoc test (SPSS 16) were used for comparison between the effects of different doses of extract with control.

## Results

### Effects of LPS on serum concentrations of cytokines in pregnant mice

As shown in figurer 1, treatment of bacterial LPS in the pregnant mice increases serum levels of pro-inflammatory cytokines, IL-1β (p<0.01), IL-6 (p<0.01) and TNF-α (p<0.001) significantly in comparison with the saline treated control group.

**Fig 1:**
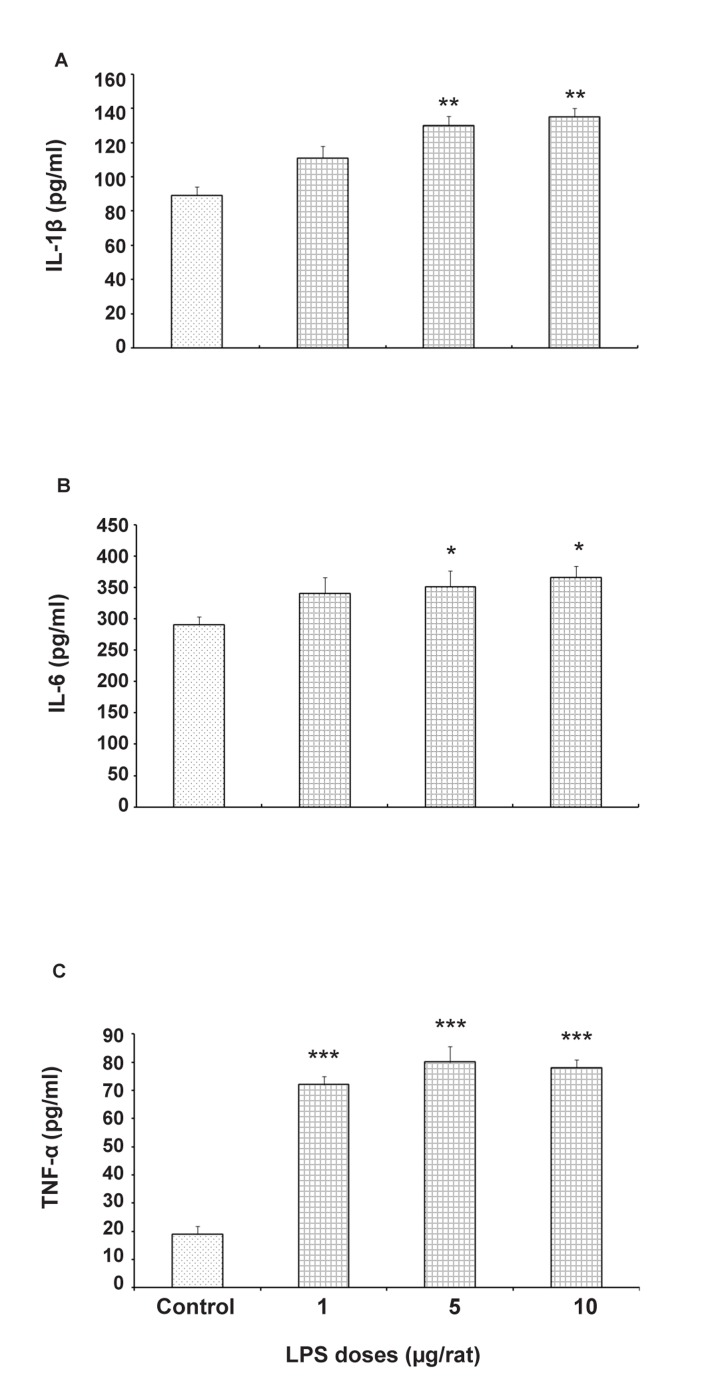
Effects of intra peritoneal injection of saline (0.05 ml/mice) or LPS (1, 5 or 10μg/Kg) on serum level of IL-1β (A), IL-6 (B) and TNF-α (C) in pregnant dams. Each bar is mean ± SE. *p<0.05, **p < 0.01 and ***p < 0.01, when compared to the saline treated group (N=7).

### Effects of prenatal LPS exposure on serum concentrations of cytokines in male offspring

**Fig 2:**
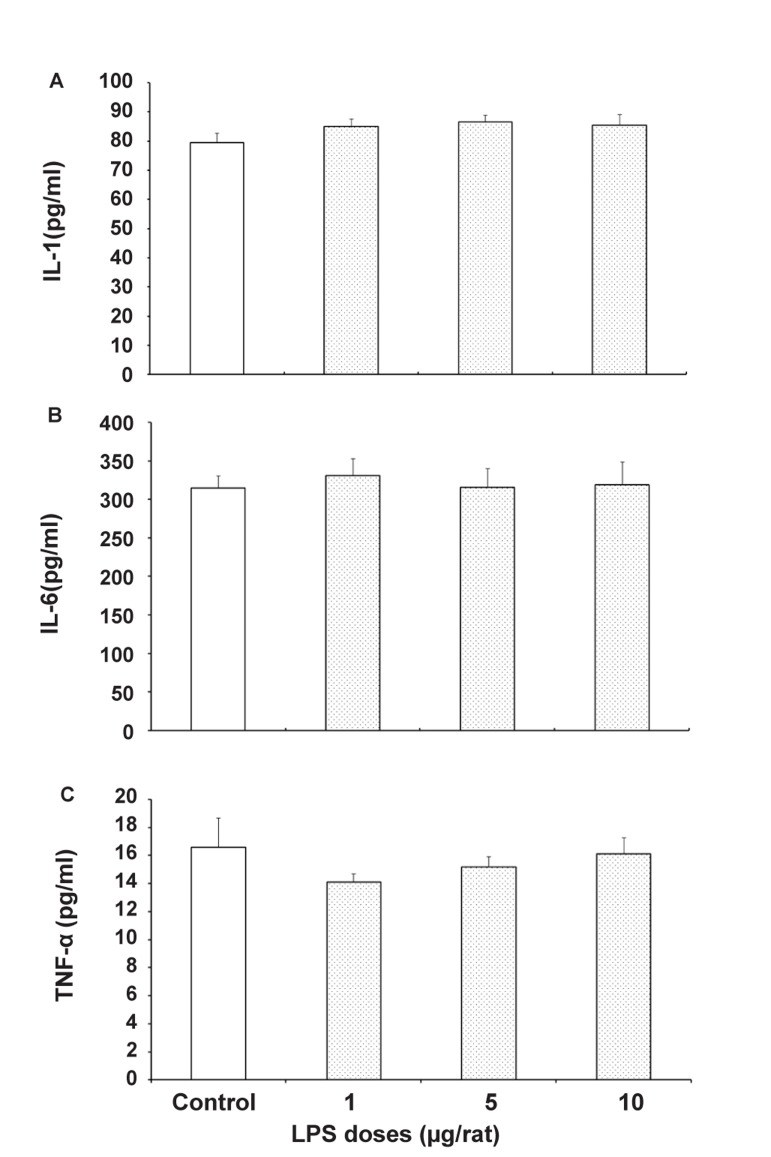
Effects of prenatal exposure of saline (0.05 ml/mice) or LPS (1, 5 or 10μg/Kg) on serum level of IL-1β (A), IL-6 (B) and TNF-α (C) in the male adult offspring. Each bar is mean ± SE (N=7).

Figure 2 shows the effects of prenatal LPS administration on serum levels of IL-1β, IL-6 and TNF-α in male offspring. As shown in the figure 2, there is no significant change in the serum cytokine levels in comparison between LPS and the saline treated control group.

### Effects of prenatal LPS exposure on sexual behaviors of offspring

The results of this research suggest that prenatal LPS exposure decreases sexual behavior components including coupling (p<0.01), following (p<0.01), mounting (p<0.01), and sniffing (p<0.001), significantly in comparison with the control group (Table 1).

### Effects of prenatal LPS exposure on pituitary-gonadal hormones of offspring

**Fig 3:**
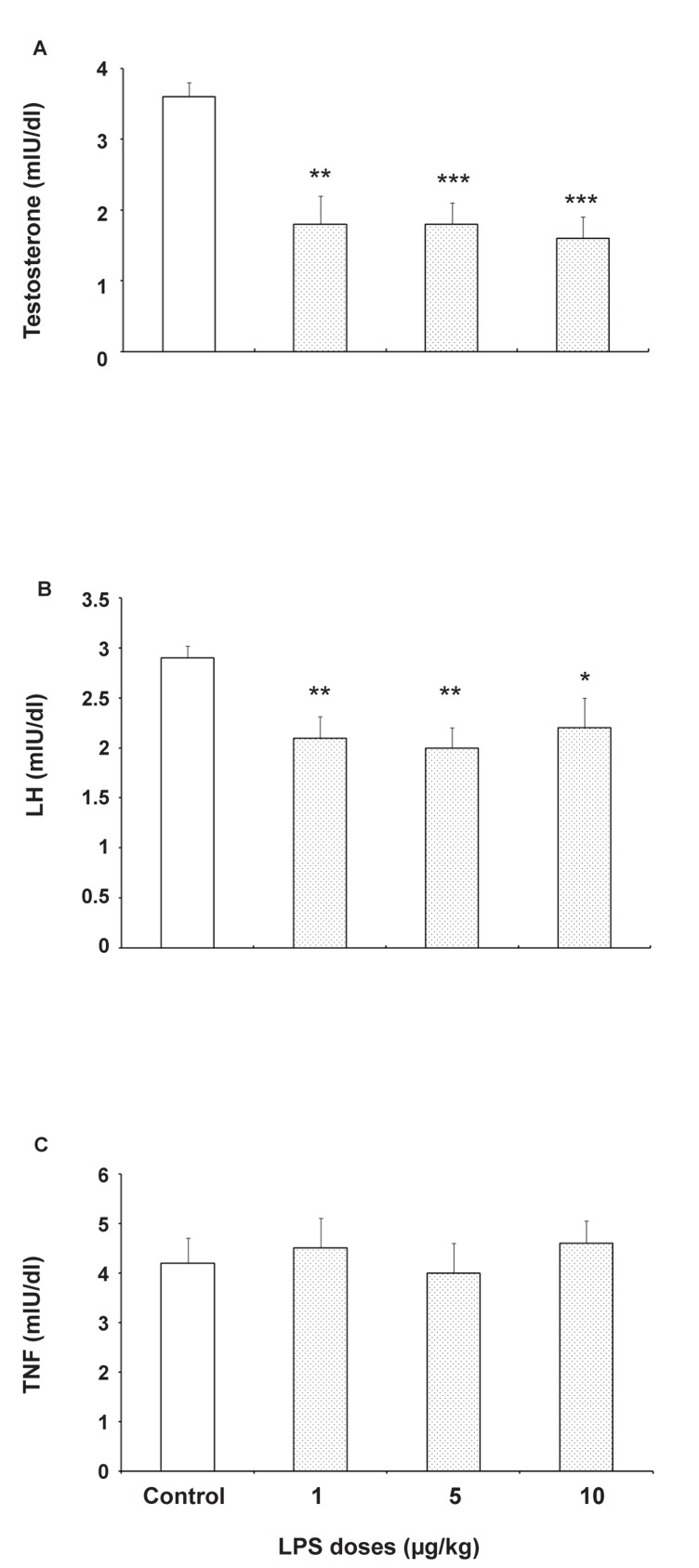
Effects prenatal exposure to saline (0.05 ml/mice) or LPS (1, 5 or 10ìg/Kg) on serum level of Testosterone (A), LH (B) and FSH (C) in adult male offspring. Each bar is mean ± SE. *p<0.05, **p<0.01 and ***p<0.01, when compared to the saline treated group (N=10).

Measuring pituitary-gonadal hormones after assessing behavioral components in prenatally LPS exposed mice showed that testosterone (p<0.001) and LH (p<0.01) concentrations of serum were significantly reduced (Fig 3). The present results also show that prenatal LPS exposure has no significant effect on serum FSH Level when compared with the control group (Fig 3).

**Table 1:** Effects of prenatal LPS exposure on behavioral components of adult Male offspring, in comparison with the control group (mean ± SE)


	Control	LPS 1(µg/rat)	LPS 5(µg/rat)	LPS 10(µg/rat)

**Number of sniffing**	16 ± 1.6	8.18± 0.78*	7.38± 0.86**	6.10± 1.1***
**Number of following**	10.78 ± 0.72	6.87± 1.5	4.81± 8.8**	4.25± 0.64**
**Number of mounting**	4.31 ± 0.6	1.7 ± 0.67	1.25± 0.44*	1.25± 0.31*
**Number of coupling**	0.8 ± 0.1	0.5 ± 1.16	0.2 ± 0.13**	0.3 ± 0.21**


*p<0.05, **p<0.01 and ***p<0.001 when compared to the saline treated group.

## Discussion

It is clear that maternal bacterial infections and immune challenges during pregnancy have distinct effects on the development of central nervous and endocrine systems in the offspring, that may affect behavior (1, 20, 21).

Results of the present study show that prenatal exposure of adult male NMRI mice with low doses of Brucella abortus LPS inhibits sexual behaviors and decreases serum level of testosterone and LH hormones. Our results also demonstrate that LPS exposure during gestation increased serum concentration of pro-inflammatory cytokines such as IL-1β, IL-6 and TNF-α. However, prenatal LPS treatment has no significant effect on serum concentrations of IL-1, IL-6 and TNF-α in the male adult offspring.

It is well known that lipopolysaccharide as a bacterial outer membrane component, induces production of several pro-inflammatory cytokines (22). Since LPS did not cross the placenta in normal conditions (11), it is possible that LPS caused it’s effects via immune mediators and inflammatory mediators, such as tumor necrosis factor (TNF-α), IL-1 and IL-6, to reach the fetus within the uterus and affect fetal developments (11, 23). However, it is also possible that the maternal exposure to infection alters pro-inflammatory cytokine levels in the fetal environment, which may have a significant impact on fetal development (11). In addition, increased pro-inflammatory cytokine levels could affect the brain of the developing fetus and may be responsible for the inhibition of the reproductive axis and its normal function in the fetuses and male newborn mice (24).

A previous study carried out by Bernardi et al. (11) showed that high dose of LPS (250 μg/kg) are able to influence some reproductive behaviors. However, the mechanism that LPS affects the sexual behavior and sexual hormone levels has not been clearly discussed yet. Studies showed that LPS treatment induces immune challenge, increases stress in animals and releases corticosterone in mice (11, 25). It is well known that maternal stress has a demasculinizing effect on male sexual behavior in mice (26-28). Bacterial LPS can activate hypothalamic-pituitary-adrenal (HPA) axis via increasing cytokine production (29, 30). Several studies have shown the activation of the HPA axis in the animals exposed to LPS. HPA activation provides an important negative feedback to cytokine production and toxicity because cytokine responses can be modulated by glucocorticoids (29, 31). Along with the inhibitory effects of HPA axis on reproduction of animals, production of pituitary-gonadal hormones have been reported in several studies (32, 33).

Increased GABAergic inhibitory activity may also contribute to the effects of prenatal stress on behavioral alteration in adulthood.

Previous studies have shown that prenatal exposure to stress and elevated levels of corticostrone affect the GABAergic system of a developing brain. Prenatal exposure to stressful environment and elevated activity of HPA-axis alters expression of GABA-A receptor subunit mRNA levels in the brain.

Prenatal exposure of the fetus to high levels of corticostrone affects mRNAs expression for glutamic acid decarboxylase (GAD) isoforms, the enzyme that converts glutamate to GABA. Stone and co-workers demonstrate that prenatal increase of corticostrone level increase GAD67 mRNA in the brain’s hippocampus. Therefore, prenatal activation of HPA axis by LPS may increase the activity of GABAergic system in the brain regions such as hippocampus that are involved in the modulation of behavior (34).

## Conclusion

Our results indicate that LPS administration on the 10th gestational day influences sexual behavior and expression of the pituitary-gonadal hormones of male offspring. Therefore, this study has identified that bacterial lipopolysaccharide exposure during pregnancy and the ensuing cytokine changes, can affect development of neural systems involved in reproduction of animals.
